# Effects of β-glucan on *Salmonella enterica* serovar Typhimurium swine colonization and microbiota alterations

**DOI:** 10.1186/s40813-023-00302-4

**Published:** 2023-02-14

**Authors:** Shawn M. D. Bearson, Julian M. Trachsel, Bradley L. Bearson, Crystal L. Loving, Brian J. Kerr, Daniel C. Shippy, Tadele G. Kiros

**Affiliations:** 1 Food Safety and Enteric Pathogens Research Unit, USDA, ARS, National Animal Disease Center, 1920 Dayton Ave, Room 1403, Ames, IA 50010 USA; 2 Agricultural Research Service Participation Program, Oak Ridge Institute for Science and Education, Oak Ridge, TN USA; 3 Agroecosystems Management Research Unit, USDA, ARS, National Laboratory for Agriculture and the Environment, Ames, IA USA; 4 Phileo by Lesaffre, North America, Milwaukee, WI USA

**Keywords:** Antibiotic alternative, β-Glucan, Microbiota, *Salmonella*, Swine

## Abstract

**Background:**

The 2017 Veterinary Feed Directive eliminated the use of medically important antibiotics for growth promotion of food animals; thus, alternative growth promoters are highly desirable by food animal producers to enhance animal health and reduce pathogen colonization, including the human foodborne pathogen *Salmonella*. β(1-3)(1-6)-d-glucan (β-glucan) is a soluble fiber with prebiotic characteristics; it has been shown to modulate immune and intestinal functions that strengthen swine resistance to health challenges such as bacterial infections when supplemented in the diets of growing pigs. The current study evaluated the effects of a β-glucan product on gut microbial community structure as well as *Salmonella* shedding and intestinal colonization.

**Results:**

Five-week-old pigs were fed a β-glucan amended diet at 500 g/ton (n = 13) or a non-amended control diet (n = 14) for three weeks, followed by inoculation of the 27 pigs with 1 × 10^9^ colony forming units of *Salmonella enterica* serovar Typhimurium strain UK1. While remaining on the respective diets, fecal samples collected at 2, 4, 7, and 16 days post-inoculation (dpi) were similar for *Salmonella* shedding counts between the two diets. At 16 dpi, *Salmonella* counts were significantly lower in the cecal contents of the β-glucan-fed pigs (P = 0.0339) and a trend towards a reduction was observed in the Peyer’s patches region of the ileum (P = 0.0790) compared to the control pigs. Pigs fed β-glucan for three weeks exhibited an increase in members of the *Clostridia* class in their fecal microbial communities, and after inoculation with *Salmonella*, several potentially beneficial microorganisms were enriched in the microbiota of β-glucan-fed pigs (*Lactobacillus, Ruminococcaceae, Prevotellaceae, Veillonellaceae, Bifidobacterium* and *Olsenella*).

**Conclusion:**

Administration of β-glucan altered the swine gut microbiome and reduced *Salmonella* colonization in the cecal contents.

**Supplementary Information:**

The online version contains supplementary material available at 10.1186/s40813-023-00302-4.

## Background

*Salmonella enterica* is a human foodborne pathogen with a high incidence of asymptomatic carriage in food animals, including swine [1–3]. *Salmonella* is recovered throughout the swine production system, including the housing environment, feed, water sources, lairage, and vectors such as insects, rodents, migratory birds, and the clothing/footwear of caretakers [4–6]. The environmentally ubiquitous nature of the > 2600 serovars of *Salmonella* combined with its commensal-like state in food animals results in an arduous pathogen to eliminate from the food chain, especially as food animal production systems expand and antibiotic usage in food animals diminishes. This dilemma necessitates the identification, development, evaluation and implementation of alternative control strategies to limit *Salmonella* in food animals, including swine.

One potential control strategy is supplementation of animal feed with β(1-3)(1-6)-d-glucan (β-glucan), a type of soluble fiber that is reported to have various beneficial properties such as immunomodulatory effects on the innate immune system [7, 8], enhanced growth performance [9], protection against infection [10], and improved intestinal tight junctions that decrease gut permeability and gut leakage [11]. These naturally occurring polysaccharides are isolated from various sources such as yeast, grain, seaweed, and algae, and are typically fermented in the animal host hindgut, providing energy substrates for the colonic epithelial cells as well as producing beneficial short chain fatty acids (SCFAs) that positively influence the microbial community of the intestinal tract [12, 13]. Stuyven et al. suggested that administration of β-glucan to weaned piglets decreased susceptibility to enterotoxigenic *Escherichia coli* (ETEC) and the supplement could be utilized as an antibiotic alternative for prevention of post-weaning diarrhea in pigs [14]. Although reports vary on the benefits of β-glucan use in *Salmonella* challenge studies of swine, poultry and cattle, reduction in transmission of *Salmonella enterica* serovar Typhimurium var Copenhagen in pigs was observed [15], as well as decreased colonization levels of the chicken ceca, liver and spleen by *Salmonella enterica* serovars Typhimurium and Enteritidis [16–18]. In the current study, evaluation of in-feed supplementation with a β-glucan product revealed significant reduction of *S*. Typhimurium in the cecal contents of pigs receiving β-glucan as well as favorable alterations in fecal microbial communities, suggesting a potential beneficial influence of the β-glucan product on the swine gut.

## Results and discussion

**Fig. 1:**
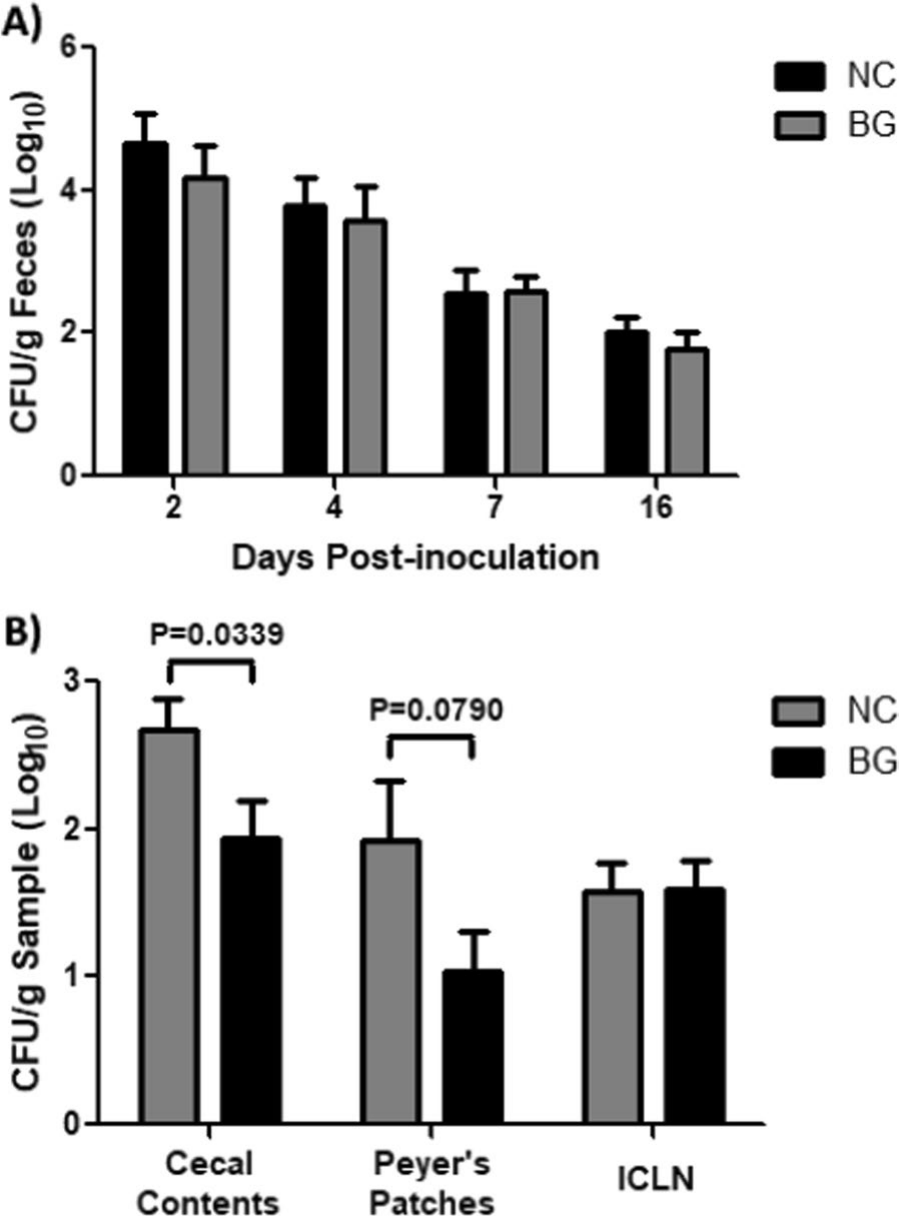
*S.* Typhimurium fecal shedding and tissue colonization following swine inoculation. At 8-weeks of age, swine administered β-glucan (500 g/ton; n = 13; BG) and pigs receiving a non-amended control diet (n = 14; NC) were intranasally inoculated with PBS containing 1 × 10^9^ CFU of wild-type *S.* Typhimurium UK1. **A** *S.* Typhimurium fecal shedding was determined at 2, 4, 7, and 16 days post-inoculation (dpi). **B** Colonization of cecal contents, ileal Peyer’s patches and ileocecal lymph nodes (ICLN) by *S.* Typhimurium was determined at 16 dpi. The error bars indicate the standard error of the mean (SEM). Differences were considered statistically significant when the P ≤ 0.05

To determine if β-glucan affects fecal microbial composition and/or *Salmonella* fecal shedding and intestinal tissue colonization, pigs receiving in-feed β-glucan (BG) for three weeks were inoculated with the UK1 strain of *Salmonella enterica* serovar Typhimurium. *Salmonella* fecal shedding was monitored at 2, 4, 7, and 16 days post-inoculation (dpi), but no significant difference was observed between the pigs receiving in-feed β-glucan compared to the pigs that consumed the non-amended control (NC) diet (Fig. 1A). Of the intestinal tissues collected at 16 dpi, a significant (P = 0.0339) reduction of *Salmonella* was observed in the cecal contents of the pigs receiving in-feed β-glucan compared to the control group (Fig. 1B). A trend towards reduction in *Salmonella* colonization was observed in the Peyer’s patches region of the ileum in the β-glucan-fed group compared to the control group, but a significant difference was not observed for *Salmonella* colonization of the ileo-cecal lymph nodes (Fig. 1B).

**Fig. 2:**
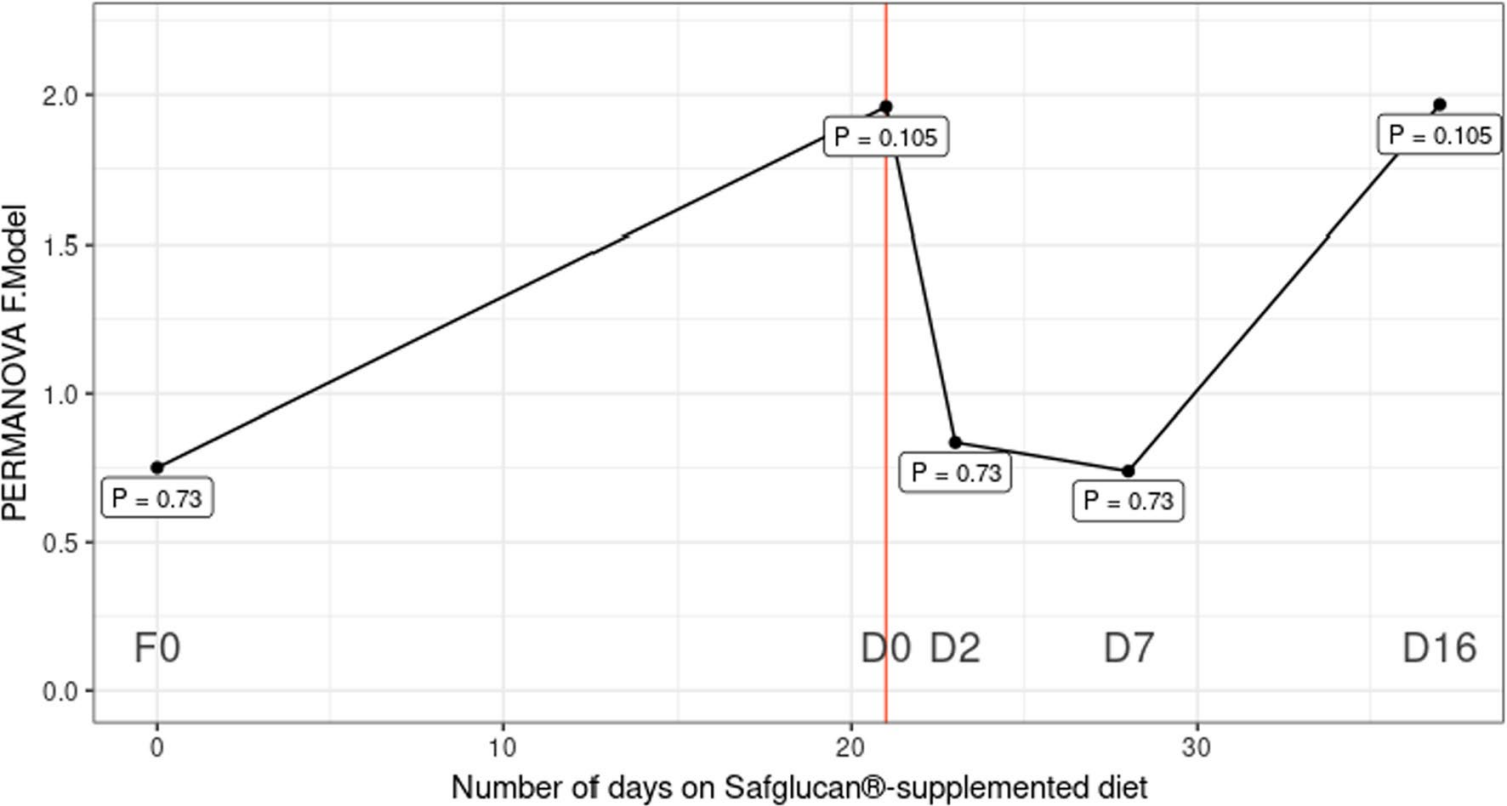
Differences in fecal bacterial community composition between swine groups (+/− β-glucan) prior to and following *Salmonella* inoculation. 16S rRNA amplicon sequencing was performed on fecal samples from pigs prior to in-feed administration of β-glucan (F0), prior to *Salmonella* inoculation (D0), and 2, 7, and 16 dpi with *Salmonella* (D2, D7, and D16, respectively). Bacterial community composition was assessed by pairwise PERMANOVA tests at the indicated timepoints. The x- and y-axis depict the time relative to β-glucan supplementation and the PERMANOVA F statistic, respectively. A greater F.model indicates a greater difference between the dietary groups. Each point represents an individual test, and FDR corrected P values are shown. *Salmonella* inoculation occurred at 21 days of β-glucan supplementation and is indicated with a vertical red line

Potential differences in the gut microbiota of pigs receiving in-feed β-glucan compared to pigs without supplementation in the context of a *Salmonella* challenge were also evaluated. Gut microbial communities were surveyed by sequencing 16S rRNA amplicons generated from fecal samples taken from the pigs prior to supplementation with β-glucan in the feed (F0), prior to *Salmonella* challenge (D0), and 2, 7, and 16 dpi with *Salmonella* (D2, D7, and D16, respectively). Overall, differences in the microbial communities of the two groups were moderate. No differences in alpha diversity were detected (Shannon index, Additional File 1: Fig. S1). Pairwise PERMANOVA tests to assess differences in community composition between dietary groups at each day (Fig. 2) suggested a difference between groups immediately before *Salmonella* challenge (PERMANOVA F = 1.9, P = 0.105). During the acute phase of *Salmonella* infection (D2 & D7), bacterial communities in both groups were similar, but by D16, a small difference in community compositions between the groups was observed (F = 1.9, P = 0.105).

**Fig. 3:**
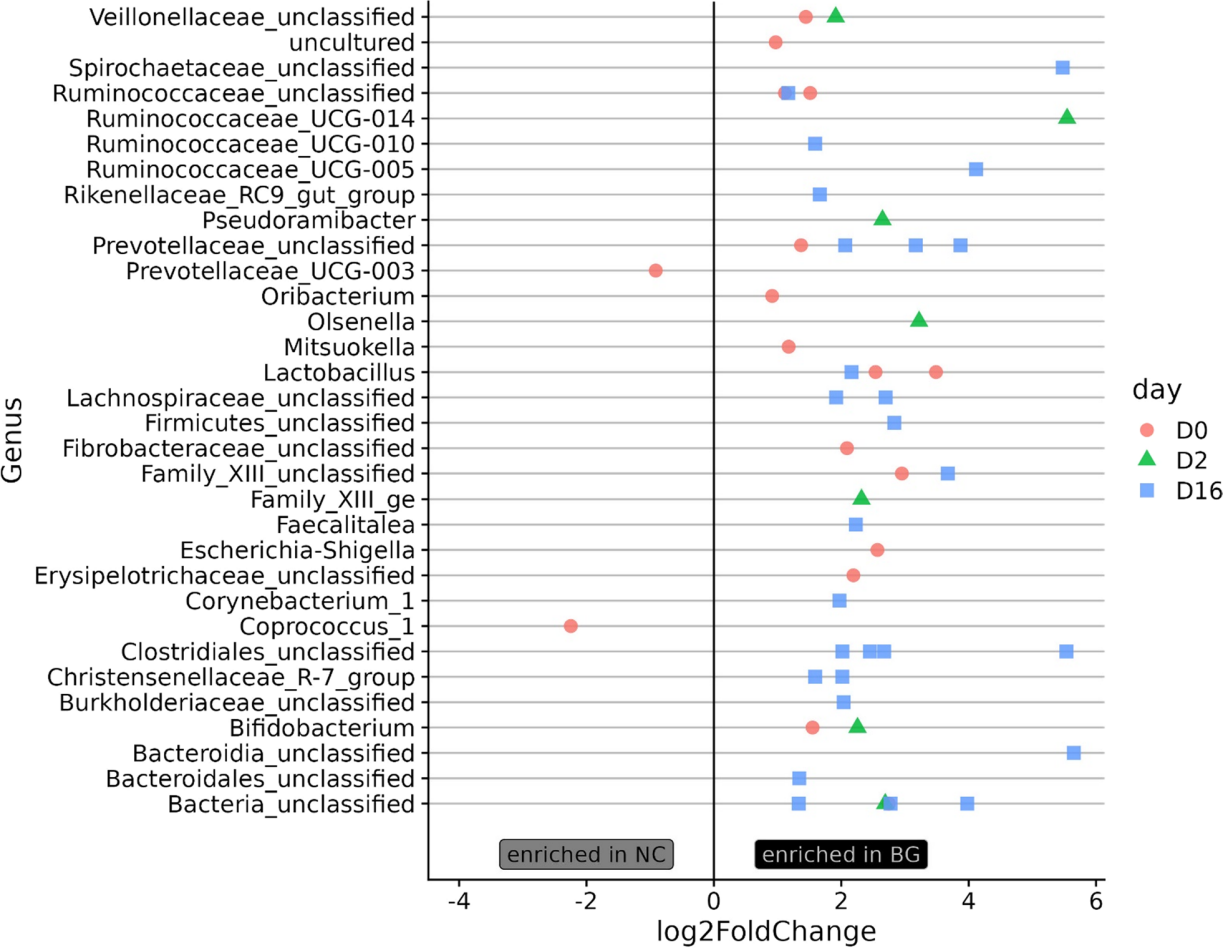
Differentially abundant OTUs in fecal samples prior to and following *Salmonella* inoculation between swine groups (+/− β-glucan). Differentially abundant OTUs were determined by DESeq2 using Wald tests with parametric fits and FDR corrected P values on fecal samples prior to *Salmonella* inoculation (D0), and at 2 (D2) and 16 (D16) dpi with *Salmonella*. OTUs with FDR P value < 0.05 and log2foldchange > 0.5 were considered differentially abundant. Each point represents a significantly differentially abundant OTU at one timepoint, and multiple points occurring on a single line indicate multiple OTUs classified to that taxa. The x- and y-axis indicate the log2foldchange (the magnitude of the difference in abundance between the two groups) and the genus the OTU was classified as according to the SILVA v132 taxonomy, respectively. A positive log2foldchange indicates the OTU was enriched in the β-glucan diet and a negative log2foldchange indicates the OTU was enriched in the non-amended control diet

Alterations in the microbiota composition were determined by comparing the differentially abundant operational taxonomic units (OTUs) between the treatment groups at each timepoint. In Fig. 3, differentially abundant OTUs that were significant between the treatment groups are presented; positive “log2FoldChange” values represent bacteria enriched in the β-glucan-fed pigs, whereas negative “log2FoldChange” values represent bacteria enriched in the pigs receiving a non-amended control diet. Detectable significant differences were observed at D0, D2, and D16, notably with many OTUs enriched in the β-glucan diet belonging to the class *Clostridia* (e.g. members of the families *Lachnospiraceae*, *Ruminococcaceae* and unclasified *Clostridiales*). These organisms are generally strict anaerobes, and their presence has been associated with a healthy gut microbiome, such as butyrate-producing bacteria that promote SCFA production [13]. In addition, OTUs from the *Bifidobacterium* and *Olsenella* genera were enriched in the β-glucan-fed pigs early in the *Salmonella* infection; our previous work associated increased abundances of OTUs classified to these genera with reduced *Salmonella* fecal shedding in swine fed a diet amended with resistant potato starch [19]. Lastly, similar bacterial enrichments were observed by other investigations of swine administered diets containing β-glucan, including *Lactobacillus* and *Ruminococcaceae* [20, 21]. Because the beneficial effects for most prebiotic diets are mediated through the gut microbiota, resident bacterial membership at the onset of diet supplementation is crucial for the success of prebiotic additives. This study contributes to the knowledge necessary to define the essential members of the microbial community for optimal utilization of β-glucan for improved gut health and pathogen reduction.

## Conclusions

In summary, three weeks of β-glucan supplementation resulted in moderate microbial community differences between the treatment groups with and without β-glucan, including a detectable increase in membership of the *Clostridia* class of bacteria in the β-glucan-fed pigs. Upon inoculation with *S*. Typhimurium, some of these potentially beneficial microorganisms remained enriched (i.e. *Lactobacillus, Ruminococcaceae, Prevotellaceae,* and *Veillonellaceae*), and an increase occurred in other community members compared to the microbiota of the pigs not receiving in-feed β-glucan. As for *Salmonella* colonization and shedding, a significantly lower level of *Salmonella* was detected in the cecal contents of pigs receiving in-feed β-glucan compared to the control pigs, and the levels of *Salmonella* in the ileal Peyer’s patches trended to be lower in the β-glucan-fed pigs; however, no significant difference was observed in *Salmonella* fecal shedding between the two treatment groups nor in the ileo-cecal lymph nodes. Overall, the data suggest that β-glucan diet supplementation resulted in modest alterations in swine gut microbiota with limited impacts on *S*. Typhimurium swine colonization and shedding.

## Materials and methods

### Swine study

Twenty-seven crossbred pigs were procured from a local farm, randomly divided into two groups, and housed in isolation barn rooms at the National Animal Disease Center (NADC) in Ames, IA. At five weeks of age, 13 pigs were administered an in-feed β-glucan product (Safglucan®, Phileo by Lesaffre, Marcq-en-Baroeul, France) at 500 g/ton, while 14 pigs received a non-amended control diet (Additional File 2: Table S1). After three weeks on the respective diets, all 27 of the eight-week-old pigs were intranasally inoculated with 1 × 10^9^ CFU of *Salmonella enterica* serovar Typhimurium strain UK1 (SB 377; [22]). Following *Salmonella* inoculation, all pigs continued on their respective diets for the remainder of the study. Individual fecal samples (taken at defecation) were collected prior to feed supplementation (F0) and at 0, 2, 4, 7 and 16 days post-inoculation (dpi). At 16 dpi, all 27 piglets were euthanized, and tissue samples (ileal-cecal lymph nodes (ICLN), Peyer’s patches of the ileum, and cecal contents) were collected for *Salmonella* detection and enumeration as previously described [23] using XLT-4 medium (Becton, Dickinson and Co., Sparks, MD) supplemented with 100% tergitol and nalidixic acid (30 µg/mL). *Salmonella* suspect colonies were evaluated on BBL™ CHROMagar™ *Salmonella* (Becton, Dickinson and Co.). For statistical analysis of fecal shedding over time, a two-way repeated measures analysis of variance (ANOVA) with a Bonferroni’s multiple comparison posttest was conducted. An unpaired *t*-test was used to determine statistical significance for tissue colonization. Differences were considered significant when the P ≤ 0.05. The Guide for the Care and Use of Laboratory Animals by the National Research Council of the National Academies was followed for all experimental procedures involving the pigs with the approval from the Animal Care and Use Committee at the USDA-ARS, National Animal Disease Center.

### Microbiota analysis

Feces collected for microbiota analysis prior to supplementation with β-glucan in the feed (F0) and at 0, 2, 7 and 16 dpi with *S*. Typhimurium were immediately placed on ice and stored at − 80 °C until DNA extraction with the Qiagen Fecal Microbiome kit (Germantown, MD). 16S rRNA gene amplicons of the V4 region were generated from feces as described in [23] in accordance with the protocol described by Kozich et. al. [24]. Amplicons were sequenced on an Illumina Miseq (La Jolla, CA) using V2 reagent kits (2 × 250 bp read lengths). Operational taxonomic units (OTUs) were generated with mothur [25] and classified using the SILVA v132 taxonomy. Global singletons were removed and samples with fewer than 2000 total reads were omitted. Samples were rarefied to an equal number of reads prior to alpha diversity and Bray–Curtis distance calculations. Unrarefied counts were used to determine differentially abundant OTUs with the DESeq2 package [26] using Wald tests with parametric fits and FDR-corrected *p*-values. The R package vegan [27] was used to assess the effect of treatment on community structure similarity through PERMANOVA tests on Bray–Curtis dissimilarities as implemented by the adonis function.

## Supplementary Information

**Additional file 1. Fig. S1**: A comparison of fecal alpha diversity (Shannon index) between the diets over time. NC=no β-glucan control pig group; BG= β-glucan-fed pig group. F0=prior to in-feed β-glucan; D0=prior to *Salmonella* inoculation; D2, D7, D16= 2, 7 and 16 days post-inoculation with *Salmonella*.

**Additional file 2. Table S1**: Feed formulation.

## Data Availability

Data requests should be directed to the corresponding author. Raw data for 16S rRNA amplicons is available through NCBI bioproject PRJNA869696.

## Abbreviations

dpi: Days post-inoculation
CFU: Colony forming units
SEM: Standard error of the mean
SCFA: Short chain fatty acids
ICLN: Ileo-cecal lymph nodes
OTU: Operational taxonomic unit
