# Supplementary material for: Effects of β-glucan on Salmonella enterica serovar Typhimurium swine colonization and microbiota alterations
Source: Porcine Health Manag. 2023 Feb 14;9:7. doi: 10.1186/s40813-023-00302-4 (PMC9926856; doi:10.1186/s40813-023-00302-4)
Supplement: Supplementary file 1 — Additional file 1. Fig. S1: A comparison of fecal alpha diversity (Shannon index) between the diets over time. NC=no β-glucan control pig group; BG= β-glucan-fed pig group. F0=prior to in-feed β-glucan; D0=prior to Salmonella inoculation; D2, D7, D16= 2, 7 and 16 days post-inoculation with Salmonella. [file 40813_2023_302_MOESM1_ESM.docx]

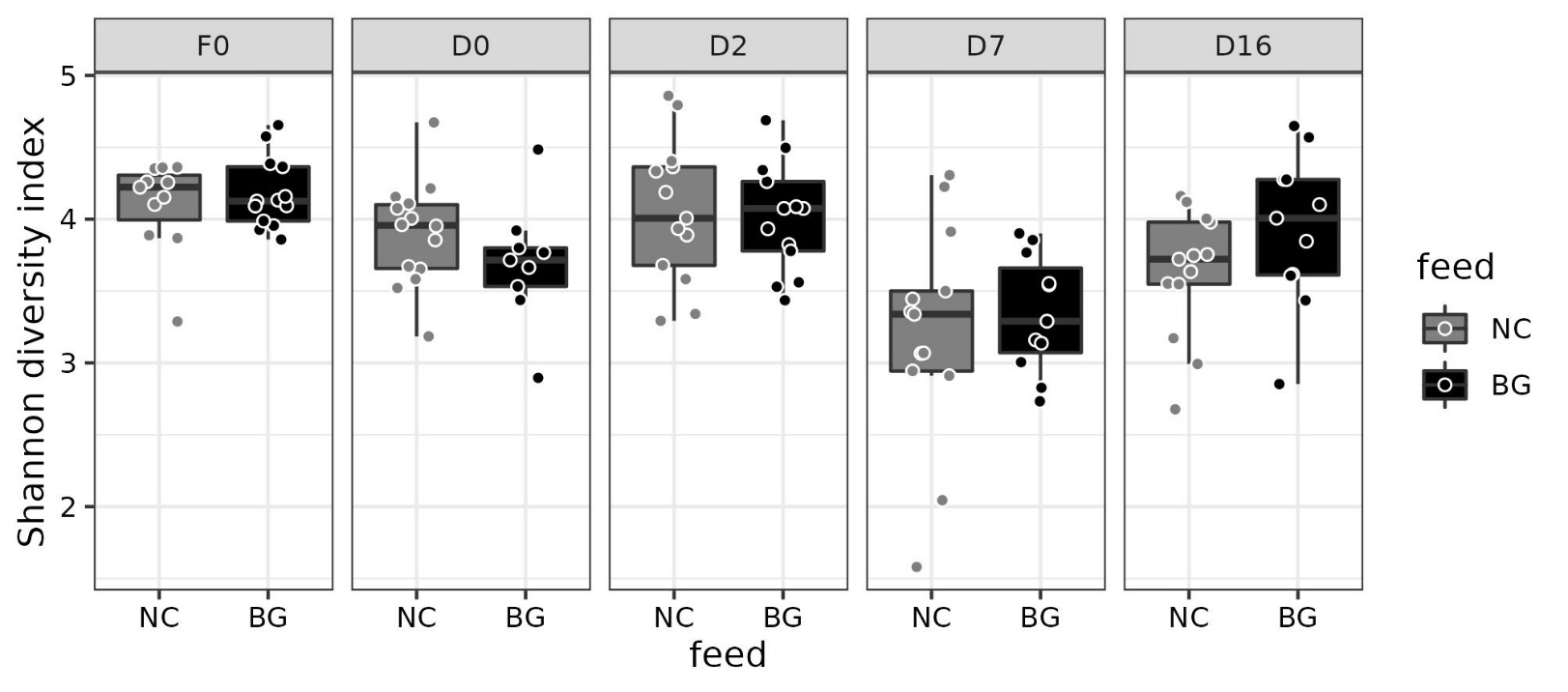
**Supplementary Figure 1, Additional File 1.** A comparison of fecal alpha diversity (Shannon index) between the diets over time. NC=no β-glucan control pig group; BG= β-glucan-fed pig group. F0=prior to in-feed β-glucan; D0=prior to *Salmonella* inoculation; D2, D7, D16= 2, 7 and 16 days post-inoculation with *Salmonella*.
