# Supplementary material for: Effects of β-glucan on Salmonella enterica serovar Typhimurium swine colonization and microbiota alterations
Source: Porcine Health Manag. 2023 Feb 14;9:7. doi: 10.1186/s40813-023-00302-4 (PMC9926856; doi:10.1186/s40813-023-00302-4)
Supplement: Supplementary file 2 — Additional file 2. Table S1: Feed formulation. [file 40813_2023_302_MOESM2_ESM.docx]

**Supplementary Table 1, Additional File 2.** Feed formulation

|  | |  | |  | | |  | |
| --- | --- | --- | --- | --- | --- | --- | --- | --- |
|  | | **Feed formulation, %** | | | | | | |
|  | | Ingredient | | | **Negative Control (NC)** | | **β-glucan (BG)** | |
|  | | Corn | | | 53.10 | | 53.07 | |
|  | | Soybean meal | | | 27.06 | | 27.06 | |
|  | | Dried whey | | | 10.00 | | 10.00 | |
|  | | Fish meal | | | 5.00 | | 5.00 | |
|  | | Soybean oil | | | 2.53 | | 2.53 | |
|  | | Monocalcium phosphate | | | 0.20 | | 0.20 | |
|  | | Limestone | | | 0.79 | | 0.79 | |
|  | | Sodium chloride | | | 0.40 | | 0.40 | |
|  | | Vitamin mix^1^ | | | 0.25 | | 0.25 | |
|  | | Trace mineral mix^2^ | | | 0.15 | | 0.15 | |
|  | | L-Lysine-HCL | | | 0.31 | | 0.31 | |
|  | | DL-Methionine | | | 0.13 | | 0.13 | |
|  | | L-Threonine | | | 0.08 | | 0.08 | |
|  | | β-glucan | | |  | | 0.0275 | |
|  | | **TOTAL** | | | **100.00** | | **100.00** | |
|  | |  | |  |  | |  | |

^1^Provided the following per kilogram of diet: vitamin A, 6,125 IU; vitamin D_3_, 700 IU; vitamin E, 50 IU; vitamin K, 30 mg; vitamin B_12_, 0.05 mg; riboflavin, 11 mg; niacin, 56 mg; and pantothenic acid, 27 mg.

^2^Provided the following per kilogram of diet: Cu (as CuSO_4_), 22 mg; Fe (as FeSO_4_), 220 mg; I (as Ca(IO_3_)_2_), 0.4 mg; Mn (as MnSO_4_), 52 mg; Zn (as ZnSO_4_), 220 mg; and Se (Na_2_SeO_3_), 0.4 mg.
